# Respiratory syncytial virus acute respiratory infection‐associated hospitalizations in preterm Mexican infants: A cohort study

**DOI:** 10.1111/irv.12708

**Published:** 2020-01-09

**Authors:** Daniela Benítez‐Guerra, Cecilia Piña‐Flores, Miguel Zamora‐López, Francisco Escalante‐Padrón, Victoria Lima‐Rogel, Ana María González‐Ortiz, Marcela Guevara‐Tovar, Sofía Bernal‐Silva, Beatriz Benito‐Cruz, Fernanda Castillo‐Martínez, Luz E. Martínez‐Rodríguez, Vianney Ramírez‐Ojeda, Nallely Tello‐Martínez, Rodrigo Lomelí‐Valdez, Jack Salto‐Quintana, Sandra Cadena‐Mota, Daniel E. Noyola

**Affiliations:** ^1^ Neonatology Department Hospital Central “Dr. Ignacio Morones Prieto” San Luis Potosí México; ^2^ Microbiology Department Facultad de Medicina Universidad Autónoma de San Luis Potosí San Luis Potosí México; ^3^ Pediatrics Department Hospital del Niño y la Mujer “Dr. Alberto López Hermosa” San Luis Potosí México

**Keywords:** acute respiratory infection, preterm infants, respiratory syncytial virus

## Abstract

**Background:**

Respiratory syncytial virus (RSV) is the leading cause of severe acute respiratory infections (ARI) in preterm infants. The incidence of RSV‐associated hospitalizations has not been defined in Mexico.

**Objectives:**

To determine the incidence of ARI‐ and RSV‐associated hospitalizations in preterm infants during the first year of life.

**Methods:**

Prospective cohort study of 294 preterm infants followed up through monthly telephone calls and routine outpatient visits. Hospitalized children were identified through daily visits to pediatric wards of participating hospitals and through telephone calls. Respiratory samples were tested for RSV by RT‐PCR.

**Results:**

Mean gestational age of participating infants was 33 weeks. Ninety‐six infants were diagnosed with bronchopulmonary dysplasia (BPD) and 17 with congenital heart disease (CHD); 11 had both conditions. There were 71 hospitalization episodes in 53 infants. Respiratory samples for RSV detection were available in 44 hospitalization episodes, and the result was positive in 16 (36.3%). At least one hospitalization for ARI was recorded in 33 of 96 participants with BPD, in seven of 17 with CHD, and 18 of 192 infants without these diagnoses. Five (71.4%) of CHD infants who required admission also had BPD. RSV‐confirmed hospitalization rates were 9.4%, 5.9%, and 2.6% for infants with BPD, CHD, and otherwise healthy preterm infants, respectively. Attributable RSV admission frequencies were estimated to be 13.6%, 16.5%, and 4.1%, respectively.

**Conclusions:**

Mexican preterm infants, particularly those with BPD, have high rates of ARI‐ and RSVassociated hospitalizations. Specific interventions to reduce the incidence of severe infections in this highrisk group are required.

## 1 INTRODUCTION

Acute respiratory infections (ARI) are a leading cause of hospitalizations and death in children <5 years of age throughout the world.^1^ Preterm infants, particularly those suffering from bronchopulmonary dysplasia (BPD), are at increased risk of severe ARI and death compared to term infants.^2^ Respiratory syncytial virus (RSV) is the most common cause of lower respiratory tract infections in children and the most common etiology of pneumonia in hospitalized infants.^3^, ^4^, ^5^ Preterm infants hospitalized with RSV infections are at high risk of requiring admission to the intensive care unit (ICU).^6^ Palivizumab is the only currently available specific intervention to reduce RSV‐associated disease.^7^ However, the high cost of this medication has limited its use in developing countries. In addition, some studies have found a lower risk for severe RSV‐associated infection than those reported in palivizumab efficacy studies, particularly in otherwise heathy preterm infants, leading to changes in the recommendations for palivizumab administration in the United States.^8^, ^9^, ^10^ However, questions still remain regarding the optimal approach for RSV prophylaxis with palivizumab, and the need for additional epidemiological data regarding the incidence of RSV hospitalizations has been identified.^11^, ^12^ The incidence of ARI‐ and RSV‐associated hospitalizations varies in different countries.^13^, ^14^, ^15^, ^16^ This could be the result of diverse factors, including social conditions, environmental factors, or healthcare systems. The incidence of severe RSV‐associated infections in preterm infants in Mexico has not been defined. Because of variability in the impact of RSV across countries, the availability of regional data is paramount in order to establish preventive measures. We carried out a prospective evaluation of infants born at <37 weeks gestation and determined the incidence of ARI‐ and RSV‐associated hospitalizations in those with and without underlying respiratory and cardiovascular disorders.

## 2 MATERIALS AND METHODS

Between 2014 and 2019, we carried out a series of studies to assess the frequency and risk factors associated with ARI hospitalizations in preterm infants. Infants born at <37 weeks of gestation and admitted to the neonatology units at Hospital Central “Dr Ignacio Morones Prieto” or Hospital del Niño y la Mujer “Dr Alberto López Hermosa” (San Luis Potosí, México) were included in these studies and followed up during the first year of life. The neonatology service at each hospital includes a neonatal intensive care unit, a neonatal intermediate care unit, and an infant growth and development unit. To allow for adequate follow‐up, only infants whose mothers' residence was within San Luis Potosí, Soledad de Graciano Sánchez, or Mexquitic de Carmona municipalities were eligible for study participation, since individuals living in these areas are expected to have access to the participating hospitals for acute medical care. Infants who died prior to discharge from the neonatal unit or those who we were unable to locate after discharge from the hospital were excluded from analysis. Palivizumab prophylaxis was not available at the participating hospitals. Prior to study enrollment, signed informed consent was obtained from the infants' parents or legal guardians. The study projects were reviewed and approved by the Research and Ethics Committees at Hospital Central “Dr Ignacio Morones Prieto” and Hospital del Niño y la Mujer “Dr Alberto López Hermosa.”

Newborn infants were enrolled in the study while hospitalized at the neonatal units at the participating hospitals. Demographical and clinical information was obtained including the presence of underlying disorders. After discharge from the hospital, follow‐up was carried out through monthly telephone calls, as well as during outpatient follow‐up visits to assess the health status of the babies. Identification of ARI‐associated hospitalizations was performed by daily visits to the pediatric wards of both hospitals in order to identify study participants who required hospital admission. Hospitalizations due to ARI that occurred at other institutions were identified through telephone communication with the infants' parents. In those patients who required admission to the hospital, clinical information was obtained from the medical records. In addition, a respiratory sample was obtained to detect the presence of RSV in patients admitted after neonatal discharge to the study hospitals. In patients that were admitted with ARI to other hospitals, respiratory samples were obtained when feasible. Viral detection was carried out by reverse transcription‐polymerase chain reaction (RT‐PCR) at the Microbiology Department, Facultad de Medicina, Universidad Autónoma de San Luis Potosí, as previously described.^6^

Acute respiratory infections‐ and RSV‐associated hospitalization incidences were assessed during the first year of life. The age at first ARI‐associated hospitalization was used for time survival analysis. The incidence per 1000 infant‐years of follow‐up was determined.

Because we could not determine if RSV was present in all ARI admissions, we calculated the RSV‐attributable admission rates in addition to the frequency of confirmed RSV infections. In order to do this, we estimated the probability of RSV infection for each admission in which a respiratory sample could not be obtained by assigning a proportion (ranging from 0 to 0.508) based on the month in which the admission occurred; this figure was obtained from results of 5074 samples that were analyzed in our laboratory from children younger than 5 years of age who were hospitalized between 2003 and 2015 in San Luis Potosí. For admissions in which RSV detection was carried out, the probability was determined to be 0 (in those negative) and 1.0 (in those with a positive result). Once this had been carried out, the RSV‐attributable admission rate was calculated adding the probability of RSV infection for all admission that occurred in the study group (and each of the sub‐groups that were analyzed) divided by the number of infants.

In addition, the characteristics of infants that required hospitalization were compared to those of infants who did not require hospitalization. BPD was defined as the use of oxygen for 28 or more days.^17^ In addition, patients with BPD were categorized as mild (those <32 weeks gestational age at birth who did not require oxygen at 36 weeks corrected gestational age or discharge, and those 32 weeks or more gestational age who did not require oxygen at 56 days of life or discharge) or moderate‐severe (those <32 weeks gestational age who required oxygen at 36 weeks corrected gestational age or at discharge, and those 32 weeks or more who required oxygen at 56 days of life or at discharge).^17^ Categorical variables were compared using the chi‐squared or Fisher's exact test, while continuous variables were compared using Student's *t* test. In addition, multivariate Cox proportional hazards analysis was carried out. Statistical analysis was carried out using SPSS for Windows, MedCalc, and Open Epi.

## 3 RESULTS

There were 49 132 births and 4071 admissions to the neonatal units at the participating hospitals during the study enrollment period. Approximately 1677 infants admitted to the neonatal units during this period were <37 weeks gestation, and their mothers' residence was either San Luis Potosí, Soledad de Graciano Sánchez, or Mexquitic de Carmona municipalities. In total, 312 newborn infants were enrolled in the follow‐up protocol. Eighteen were excluded due to death within the neonatal unit or lack of follow‐up after discharge from the hospital. The final study group included 294 infants: 128 (43.5%) were female and 166 (56.5%) were male. The mean gestational age and birthweight were 33 weeks and 1668 g, respectively. The most common diagnoses during admission to the neonatology units were respiratory distress syndrome (n = 188; 63.9%), neonatal sepsis (n = 126; 42.9%), and neonatal pneumonia (n = 88; 29.9%). Ninety‐six infants developed BPD, and in 17, congenital heart disease was diagnosed; eleven of them had both diagnoses. In total, 102 participants had BPD or congenital heart disease. Heart defects identified in the study population were the following: patent foramen ovale in seven (other abnormalities were present in two of them: pulmonary artery branch stenosis [n = 1] and patent ductus arteriosus‐associated coarctation of the aorta [n = 1]); ventricular septal defect in six (other abnormalities were present in five of them: patent foramen ovale [n = 3]; atrial septal defect [n = 1], and pulmonary artery branch stenosis [n = 1]); single atrium, transposition of the great vessels, and right aortic arch in one; coarctation of the aorta in one; pulmonary artery branch stenosis in one; and pulmonary artery hypertension with tricuspid insufficiency in one. In total, nine of them also had patent ductus arteriosus. Two patients were treated with diuretics, and two patients had corrective surgery; the other 13 patients did not require any treatment.

The mean hospitalization duration after birth in the complete study group was 31.4 days. Follow‐up was carried out for a mean of 10.4 months (range 1‐12 months), and 212 (72.1%) completed the 12‐month follow‐up schedule. Fifty‐three (18%) of the 294 participating infants had at least one admission to the hospital (range 1‐5 hospitalizations per patient). Overall, there were 74 admissions to the hospital due to ARI; three patients were readmitted with a respiratory illness within 7 days from a previous ARI hospitalization; for these instances, both episodes were considered as a single hospitalization. Therefore, the total number of hospitalizations in the study was 71. The overall hospitalization rate was 278 episodes per 1000 child‐years of follow‐up. Survival analysis taking into account patients lost to follow‐up showed that by 1 year of age, up to 22% of infants required admission due to an ARI. The cumulative incidence of ARI hospitalization was notably higher in infants with BPD compared to those preterm infants without BPD.

The characteristics of infants who required at least one ARI‐associated hospitalization and those who were not hospitalized due to ARI were compared. Infants who required ARI hospitalization during follow‐up were diagnosed with neonatal pneumonia and patent ductus arteriosus more frequently than those who did not require hospitalization (47.2% vs 26.1% and 35.8% vs 10.4%, respectively). BPD and congenital heart disease diagnoses were also more frequent among infants who were hospitalized compared with those who were not (62.3% vs 26.1% and 13.2% vs 4.1%, respectively). At least one hospitalization for ARI was required in 33 (34.4%) of the 96 infants with BPD, in 7 (41.2%) of the 17 with congenital heart disease, and in 35 (34.3%) of the 102 patients with either or both diagnoses; in contrast, 18 (9.4%) of 192 infants without these diagnoses required admission to the hospital due to ARI. The seven infants with congenital heart disease diagnosis who required hospitalization were among those that did not require treatment; however, five of them (71.4%) also had BPD. No significant association was observed between gestational age and birthweight and ARI admission; however, children who were admitted due to ARI had required more ventilator support during the neonatal period and had longer NICU stays than those who were not admitted for ARI. Multivariate Cox proportional hazards analysis showed an increased ARI hospitalization risk for infants with patent ductus arteriosus (hazard ratio [HR] 2.36, 95% CI 1.19‐4.68; *P* = .01) and BPD (HR 4.04; 95% CI 1.78‐9.13; *P* < .001; Table 1).

**Table 1:** Proportional hazards analysis of ARI‐associated hospital admissions during the first year of life

	Hazard ratio	95% confidence interval	*P*
Sex			
Female	(1.00)		.67
Male	0.88	(0.504‐1.55)	
Gestational age at birth (weeks)	1.13	(0.95‐1.33)	.17
Birthweight (g)	1.00	(0.99‐1.001)	.49
Diagnoses during admission in the neonatology unit			
Respiratory distress syndrome	0.97	(0.49‐1.91)	.94
Neonatal sepsis	1.13	(0.60‐2.12)	.71
Neonatal pneumonia	1.62	(0.88‐3.01)	.12
Patent ductus arteriosus	2.36	(1.19‐4.68)	.01
Congenital heart disease	1.42	(0.60‐3.33)	.43
Bronchopulmonary dysplasia	4.04	(1.78‐9.13)	<.001
Interventions during admission in the neonatology unit			
Oxygen supplementation	4.66	(0.60‐36.16)	.14
Mechanical ventilation	1.16	(0.57‐2.34)	.68
Hospitalization duration (days)	0.99	(0.98‐1.01)	.52
Received breast milk	0.81	(0.39‐1.71)	.59

In addition to Hospital Central “Dr Ignacio Morones Prieto” and Hospital del Niño y la Mujer “Dr Alberto López Hermosa,” patients were admitted due to ARI to several other hospitals within the metropolitan area of San Luis Potosí. Therefore, respiratory samples were not available for RSV detection in all hospitalizations. A respiratory sample was available for RSV testing in 44 (61.9%) of the 71 admission episodes, and a positive result for this virus was obtained in 16 (36.4%) of them. Overall, 14 (4.8%) participants had at least one RSV‐associated hospitalization (RSV was detected in two episodes in two patients). The RSV‐confirmed hospitalization rates were 9.4%, 5.9%, and 2.6% for infants with BPD, congenital heart disease, and otherwise healthy preterm infants, respectively. Overall, the hospitalization rate for RSV‐confirmed ARI was 62.6 per 1000 child‐years of follow‐up.

The characteristics during the neonatal period of infants admitted with confirmed RSV infection were compared to those not admitted with RSV. Infants admitted to the hospital for whom a respiratory sample was not available for RSV testing were excluded from this analysis, since infection with this virus could not be ruled out. The main characteristics during the neonatal period associated with RSV admission were diagnosis of respiratory distress syndrome (present in 100% of infants with RSV‐associated hospitalization), neonatal sepsis (present in 78.6%), and BPD (present in 64.3%). Multivariate Cox proportional hazards analysis was carried out, but no independent association was found between any of these variables and RSV‐associated admission (Table 2).

**Table 2:** Proportional hazards analysis of RSV‐associated hospital admissions during the first year of life

	Hazard ratio	95% CI	*P*
Sex			
Female	(1.00)		.47
Male	0.91	0.71‐1.17	
Gestational age at birth (weeks)	0.91	0.91‐1.08	.91
Birthweight (g)	1.00	0.99‐1.00	.85
Diagnoses during admission in the neonatology unit			
Respiratory distress syndrome	0.95	0.69‐1.28	.72
Neonatal sepsis	0.92	0.70‐1.22	.57
Neonatal pneumonia	1.01	0.73‐1.39	.97
Patent ductus arteriosus	0.89	0.57‐1.39	.62
Congenital heart disease	0.77	0.41‐1.44	.41
Bronchopulmonary dysplasia	1.02	0.66‐1.56	.93
Interventions during admission in the neonatology unit			
Oxygen supplementation	0.99	0.67‐1.48	.99
Mechanical ventilation	1.19	0.79‐1.79	.39
Hospitalization duration (days)	1.00	0.99‐1.01	.61
Received breast milk	1.02	0.69‐1.49	.91

**Figure 1:**
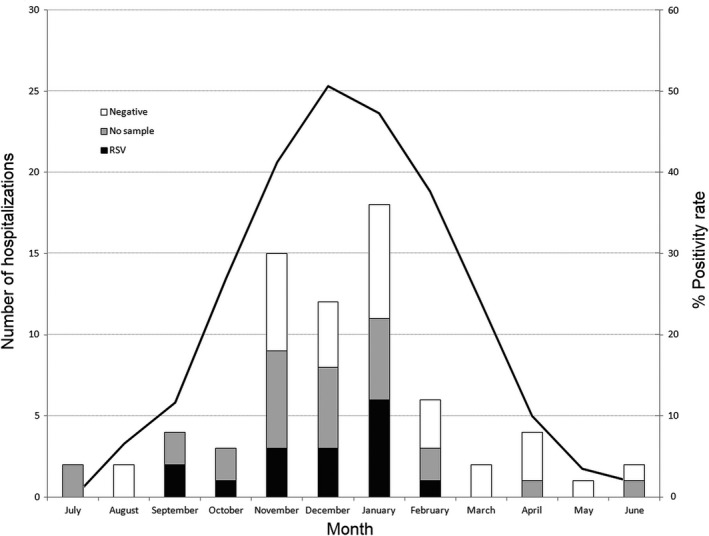
Acute respiratory infections hospital admission episodes in study participants, according to month of occurrence and proportion of RSV detection in 5074 children <5 y of age admitted with ARI

Because we were not able to obtain a sample for viral detection in all episodes, the recorded number of RSV hospitalizations corresponds to a minimum incidence. In order to estimate an RSV hospitalization incidence closer to the actual incidence, we determined the probability that a hospitalization in which no sample was obtained might have been caused by RSV as described in the Methods section. The number of ARI hospital admission episodes in study participants during each month and the proportion of RSV detection in 5074 children <5 years of age hospitalized with ARI are shown in Figure 1. These results were used to estimate the RSV‐attributable admission rate (Table 3). Overall, the RSV‐attributable ARI admission rate was 94.1 per 1000 child‐years of follow‐up.

**Table 3:** Acute respiratory infections, RSV‐confirmed, and RSV‐attributable admission rates in preterm infants

Infant characteristic	Number of subjects	Number admitted with ARI	Number admitted with confirmed RSV	Attributable RSV admissions^a^
All participants	294	53 (18%)	14 (4.8%)	22 (7.5%)
Gestational age at birth				
<28 wk	10	2 (20%)	0 (0%)	0.5 (5%)
28‐31 wk	63	16 (25.4%)	3 (4.8%)	4.7 (7.5%)
32‐34 wk	142	26 (18.3%)	9 (6.3%)	13.9 (9.8%)
35‐36 wk	79	9 (11.4%)	2 (2.5%)	2.9 (3.7%)
BPD	96	33 (34.4%)	9 (9.4%)	13.1 (13.6%)
Mild BPD	34	10 (29.4%)	5 (14.7%)	5.9 (17.3%)
Moderate‐severe BPD	62	23 (37.1%)	4 (6.4%)	7.2 (11.6%)
Congenital heart disease	17	7 (41.2%)	1 (5.9%)	2.8 (16.5%)
BPD or congenital heart disease	102	35 (34.3%)	9 (8.8%)	14.1 (13.8%)
Preterm without BPD or congenital heart disease	192	18 (9.4%)	5 (2.6%)	7.9 (4.1%)
Month of birth				
October‐March	173	28 (16.2%)	8 (4.6%)	12.8 (7.4%)
April‐September	121	25 (20.7%)	6 (4.9%)	9.2 (7.6%)

a RSV‐attributable admissions were estimated by adding the number of confirmed infections and the estimated probability of RSV infection in those infants who did not have a sample available for viral testing.

Neither ARI nor RSV admission rates showed an apparent association with the month of birth. BPD and congenital heart disease were associated with high hospitalization rates compared to preterm infants without these risk factors; however, severity of BPD did not appear to affect the risk of hospital admission (Table 3). Overall, infants with BPD or congenital heart disease required hospitalization approximately 3.5 times more frequently than those without these risk factors.

We also analyzed the cumulative proportion of ARI‐ and RSV‐confirmed infections according to the age of participants at the time of admission, as well as taking into account the month in which the admission occurred, considering that the beginning of the RSV season usually occurs in October in our city. The cumulative proportions of admissions are shown in Figure S1. Overall, 87.5% of confirmed RSV admissions in the study participants occurred between October and March, and 75% of these occurred in infants <6 months of age.

The main clinical characteristics observed during the hospitalizations are shown in Table S1. The most common discharge diagnosis was pneumonia (47 episodes; 66.2%), followed by bronchiolitis (19 episodes; 26.8%). Forty‐two (59%) admissions occurred during the first 6 months of life. Hospitalizations caused by RSV occurred in younger infants than those in which this virus was not detected (mean of 3.7 months compared to 5.9 months, respectively; *P* = .02). The signs and symptoms on admission were similar between episodes in which RSV was detected compared to those in which this virus was not identified, with the exception of cyanosis, which was more common in infants with RSV infection. Eight infants were admitted to the ICU, and seven required mechanical ventilation.

Five patients died during the study period, and two of these were associated with an ARI hospitalization. RSV was detected in one of them, while in the other infant no sample was available for viral testing. Overall, ARI and RSV were associated to 40% and 20% of the recorded deaths.

## 4 DISCUSSION

Acute respiratory infections continue to be a significant cause of morbidity and mortality worldwide. The World Health Organization estimated that 16% of deaths in children <5 years of age were due to ARI in 2015.^18^ In Mexico, ARI are responsible for approximately 5.9% of deaths in children <5 years of age.^19^ However, the relative contributions of specific pathogens and underlying conditions to severe cases of ARI have not been defined. Preterm born infants, which constitute 10.6% of all births globally, have a higher risk for severe ARI than infants born at term.^20^, ^21^ In addition, infants with complications secondary to prematurity, particularly BPD, have the highest risk for severe infection and mortality. Therefore, interventions to reduce the impact of ARI in these populations are warranted. The availability of information regarding the incidence and risk factors associated with these infections is of value in order to establish and evaluate preventive programs.

While it is well established that prematurity, BPD, and congenital heart diseases are risk factors for ARI‐ and RSV‐associated hospitalizations, the impact of these conditions on infant health varies in different countries.^22^, ^23^ In addition, healthcare utilization may vary also in time. Some studies carried out in the United States show lower hospitalization rates in preterm infants than those reported in previous analyses.^10^ Also, the incidence of RSV‐associated hospitalization varies in studies carried out in different countries.^24^, ^25^, ^26^ In a systematic review that assessed the incidence of RSV hospitalization in healthy preterm infants 29‐35 weeks of age, a wide variation of in hospitalization rates (2.3%‐10%) was observed.^16^ This variability may be a reflection of differences in environmental conditions, social factors, healthcare systems, or genetic polymorphisms, among others possible explanations. As such, up‐to‐date information regarding the impact of RSV and other respiratory viruses in different countries is required in order to define preventive strategies that are appropriate for each region.

Despite the relevance of ARI and RSV as a cause of hospitalizations and mortality, in Mexico, the incidence of hospitalizations in preterm infants due to these conditions has not been previously established. In this study, we determined that 18% of participants required at least one ARI‐associated hospitalization during the first year of life. An RSV‐confirmed hospitalization occurred in 4.8% of infants, and the RSV‐attributable hospitalization frequency was 7.5%. The proportion of infants with BPD or congenital heart disease who required at least one hospitalization was notably higher: 34.4% required at least one hospitalization, and the estimated RSV‐attributable admission frequency was 13.8%.

The only study carried out previously in Mexican children that has been published was done as part of a multi‐national analysis of infants who received palivizumab.^27^ Because no separate data for participants from each country are provided, and because all children received palivizumab, it is difficult to compare our results with those reported in that study.

A limitation of our study is that a respiratory sample for RSV testing could not be obtained in all ARI hospitalization episodes. Of note, the healthcare system in Mexico is rather complex and includes three large public health systems (Ministry of Health hospitals and clinics, Mexican Social Security Institute [IMSS], and Institute for Social Security and Services for State Workers [ISSSTE]), other healthcare institutions (such as the Ministry of Defense hospitals), as well as diverse private hospitals and clinics. Admissions for ARI after discharge from the neonatology units were registered in other hospitals and clinics in addition to the two hospitals where the study was carried out. As a result, timely identification of admissions for sample collection was not always feasible.

Overall, infants with BPD showed a very high frequency of ARI‐ and RSV‐associated hospitalizations, regardless of gestational age. This contrasts with current recommendations for palivizumab use which suggest this monoclonal antibody should be used only for infants with BPD and gestational age <32 weeks.^28^ As such, our data indicate that, in our population, all infants with BPD are at increased risk for severe RSV infection and, therefore, prophylaxis with palivizumab should be considered. On the other hand, the number of infants with hemodynamically significant heart disease in our study was small, and the majority of children with congenital heart defects also had BPD. Therefore, we did not have sufficient data to assess the independent risk for ARI‐ and RSV‐associated hospitalizations in this group of children.

In our study, we did not include full‐term infants. As a result, we cannot determine if the incidence rates in preterm infants are higher than those of term infants. However, the incidence or RSV‐confirmed (62.6 per 1000 child‐years) and RSV‐attributable admissions (94.1 per 1000 child‐years) in our study are similar to those reported worldwide during the first year of life in preterm infants (63.85, 95% CI 37.52‐109.7, hospitalizations per 1000 children per year).^29^ In contrast, the global incidence of RSV hospitalizations in all children <1 year of age was estimated at 19.19 (95% CI 15.05‐24.48) per 1000 children per year. As such, our data are consistent with a higher incidence of ARI‐associated hospitalizations in preterm infants.

In summary, we have determined the frequency of ARI‐ and RSV‐associated hospitalizations in Mexican preterm infants without palivizumab prophylaxis. Eighteen percent of participants required at least one admission to the hospital, and the overall ARI hospitalization incidence was 278 episodes per 1000 child‐years of follow‐up. ARI‐ and RSV‐associated hospitalizations were significantly higher in infants with BPD. These results support the need to establish a national program to reduce the incidence and severity of RSV infections in all infants with this underlying condition.

## CONFLICTS OF INTEREST

D E. Noyola has participated as a member of the speakers' bureau of AbbVie and speakers' bureau and advisory board for Sanofi Pasteur. All other authors report no potential conflicts of interest.
