# Supplementary figures and images for: Respiratory syncytial virus acute respiratory infection‐associated hospitalizations in preterm Mexican infants: A cohort study
Source: Influenza Other Respir Viruses. 2020 Jan 9;14(2):182–8. doi: 10.1111/irv.12708 (PMC7040972; doi:10.1111/irv.12708)

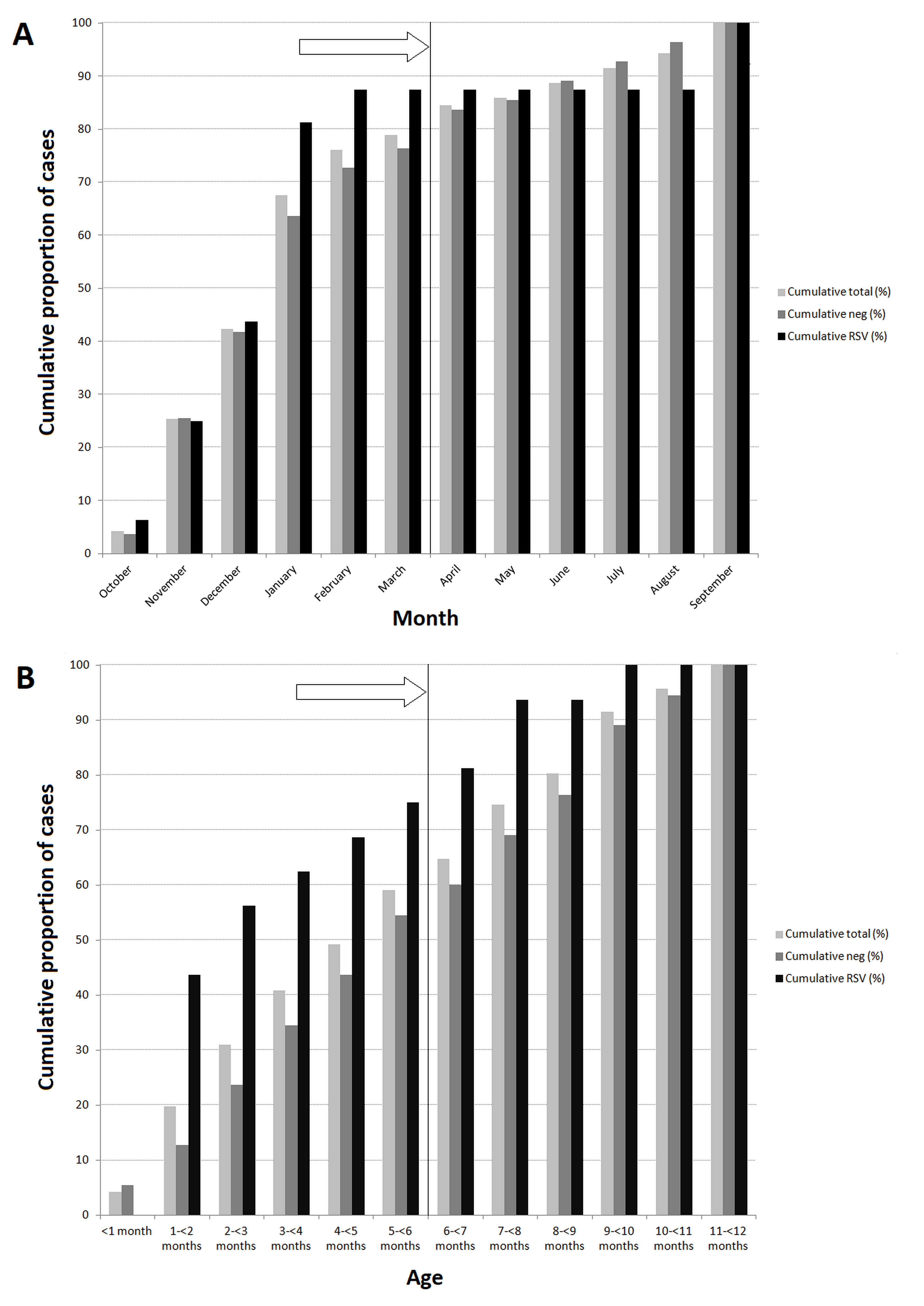

Supplement: Supplementary file 1 [file IRV-14-182-s001.tif]
