# Supplementary material for: Respiratory syncytial virus acute respiratory infection‐associated hospitalizations in preterm Mexican infants: A cohort study
Source: Influenza Other Respir Viruses. 2020 Jan 9;14(2):182–8. doi: 10.1111/irv.12708 (PMC7040972; doi:10.1111/irv.12708)
Supplement: Supplementary file 2 [file IRV-14-182-s002.docx]

| **Supplementary Table 1.** Characteristics of 71 hospitalization episodes associated to ARI in preterm infants | | | |
| --- | --- | --- | --- |
|  | RSV detected  (n=16) | Negative or no sample available (n=55) † | P |
| Sex Female  Male | 5 (31.25%)  11 (68.75%) | 27 (49.1%)  28 (50.9%) | 0.21 |
| Age at admission (months) | 3.7 (2.7) | 5.9 (3.34) | 0.02 |
| Symptoms |  |  |  |
| Cough | 15 (93.75%) | 50 (91.7%) | 0.99 |
| Respiratory distress | 10 (62.5%) | 43 (78.2%) | 0.34 |
| Rhinorrea | 10 (62.5%) | 35 (63.6%) | 0.93 |
| Nasal congestion | 8 (50%) | 17 (30.9%) | 0.16 |
| Fever | 4 (25%) | 24 (43.6%) | 0.18 |
| Signs |  |  |  |
| Cyanosis | 10 (62.5%) | 14 (25.9%)‡ | 0.007 |
| Intercostal retractions | 9 (56.25%) | 30 (56%)‡ | 0.96 |
| Thoracoabdominal dysociation | 6 (37.5%) | 22 (40.7%)‡ | 0.82 |
| Wheezing | 9 (56.25%) | 30 (56%)‡ | 0.96 |
| Crackles | 8 (50%) | 36 (66.7%)‡ | 0.23 |
| Stridor | 0 (0%) | 3 (5.6%)‡ | 0.91 |
|  |  |  |  |
| Oxygen requirement | 14 (87.5%) | 53 (96.4%) | 0.43 |
| ICU admission | 3 (18.75%) | 5 (9.1%) | 0.51 |
| Mechanical ventilation | 3 (18.75%) | 4 (7.3%) | 0.37 |
| Hospitalization duration (days) | 10.13 (7.2) | 8.24 (9.9) | 0.48 |
| Death | 1 (6.25%) | 1 (1.8%) | 0.8 |
|  |  |  |  |
| Discharge diagnosis |  |  |  |
| Pneumonia | 10 (62.5%) | 37 (67.3%) | 0.72 |
| Bronchiolitis | 5 (31.25%) | 14 (25.4%) | 0.87 |
| Other§ | 1 (6.25%) | 4 (7.3%) | 0.99 |
| † Includes 28 RSV-negative admissions and 27 admissions without available sample  ‡ Data missing from one case  § Reactive airways disease (3 cases), whooping cough (1 case), upper respiratory infection + apnea (1 case) | | | |
